# Psychometric properties of the pain stages of change questionnaire as evaluated by rasch analysis in patients with chronic musculoskeletal pain

**DOI:** 10.1186/1471-2474-15-95

**Published:** 2014-03-19

**Authors:** Cecilie Røe, Elin Damsgård, Terese Fors, Audny Anke

**Affiliations:** 1 Department of Physical Medicine and Rehabilitation, Oslo University Hospital Ullevål, Pb4956 Nydalen 0429, Oslo, Norway; 2 Faculty of Medicine, University of Oslo, Oslo, Norway; 3 Department of Rehabilitation, University Hospital of North Norway, Tromsø, Norway; 4 Faculty of Health Sciences, Institute of Clinical Medicine, University of Tromsø, Tromsø, Norway; 5 Department of Health and Care Sciences, University of Tromsø, Tromsø, Norway

**Keywords:** Musculoskeletal pain, Pain stages of change, Self-management, Rasch analysis

## Abstract

**Background:**

Our objective was to evaluate the measurement properties of the Pain Stages of Change Questionnaire (PSOCQ) and its four subscales Precontemplation, Contemplation, Action and Maintenance.

**Methods:**

A total of 231 patients, median age 42 years, with chronic musculoskeletal pain responded to the 30 items in PSOCQ. Thresholds for item scores, and unidimensionality and invariance of the PSOCQ and its four subscales were evaluated by Rasch analysis, partial credit model.

**Results:**

The items had disordered threshold and needed to be rescored. The 30 items in the PSOCQ did not fit the Rasch model Chi- square item trait statistics. All subscales fitted the Rasch models. The associations to pain (11 point numeric rating scale), emotional distress (Hopkins symptom check list v 25) and self-efficacy (Arthritis Self-Efficacy Scale) were highest for the Precontemplation subscale.

**Conclusion:**

The present analysis revealed that all four subscales in PSOCQ fitted the Rasch model. No common construct for all subscales were identified, but the Action and Maintenance subscales were closely related.

## Background

Chronic musculoskeletal pain is a major cause of sickness absence and disability [1] and represents a great challenge to society. Self-management is increasingly emphasised as part of a multidisciplinary treatment program [2]. However, clinical experience indicates that some patients participating in these programs improve, whereas others do not benefit [3]. How motivated the patient is to engage in treatment recommendations may affect the way a person carries out the program. It may also determine the outcome and influence the choice of clinical approach [4,5]. Hence, there is a need for clinical evaluation tools to determine the orientation and motivation of the patient towards a self-management approach.

Kerns and colleagues proposed a model for conceptualising the process of adopting a self-management approach to chronic pain, and developed the Pain Stages of Change Questionnaire (PSOCQ) [6]. PSOCQ is based on the Transtheoretical Model (TTM) for behavioural change. A central construct of the model is the Stages of Change [7]. The questionnaire measures the extent to which an individual accepts personal responsibility for pain control and is considering making behavioural changes to cope with the pain. It is composed of four scales: Precontemplation, Contemplation, Action and Maintenance that correspond theoretically to different stages of change. A central challenge with PSOCQ is the problem of assigning patients to reliable stage groups. Studies have pointed out the relative lack of difference between persons identified as being in different stages [6,8,9]. The stages also overlap regarding correlation to other characteristics of the patients like pain, emotional distress and coping [10].

Three lines of research have been adopted to approach this challenge. The most used approach has been to adopt the participant’s single highest subscale score [8]. Alternative versions of PSOCQ have been developed and evaluated to attempt to improve the discriminant validity of the scales [11,12]. The third alternative has been to create individual profiles of scores based on scoring profiles on the subscales [10,13].

The factor structure of PSOCQ has been explored revealing from two to four factors [9,11,14]. Most consistently there is a high correlation between Action and Maintenance [14]. However, the measurement properties of the subscales regarding internal consistency, possible redundancy of items and invariance across subgroups of patients have not been investigated. Rasch analysis provides the information of these properties, as well as the unidimensionality of a measurement [15]. Invariance of a measurement or subscale regarding important characteristics of the patients is also a prerequisite to obtain sumscores across patient subgroups [16]. Age, gender and education are important factors that may influence measurement of readiness to self-management life [17,18].

Although no measurement provide the possibility to evaluate concurrent validity directly, the relationship to pain, coping and emotional status is considered important to evaluate aspects of self-management [14].

Our objective was to evaluate the PSOCQ and its four subscales by Rasch analysis in order to answer the following questions:

– Does PSOCQ represent a unidimensional construct within the Transtheoretical Model for behavioural change?

– Do the four subscales represent separate unidimensional constructs of Precontemplation, Contemplation, Action and Maintenance?

– Are PSOCQ and its subscales invariant to subgroups of patients across important factors, such as age, gender and education?

– To which extent are the subscales associated to pain, emotional distress and self-efficacy?

## Methods

### Participants

Participants were recruited from the “Neck and Back” unit at the University Hospital of North Norway, Department of Physical Medicine and Rehabilitation in the period from October 2005 through October 2006. The clinic receives patients referred from primary healthcare with various musculoskeletal pain conditions. Inclusion criteria were: first time visit, understanding and speaking the Norwegian language, and age between 18 and 67 years. Patients with suspected malignant diseases were excluded. Approximately 5% of the referred patients did not meet the inclusion criteria. Of 549 eligible subjects, 263 subjects (48%) gave informed consent, and one subject was excluded due to suspected malignancy. Thirty-two responders were excluded due to lack of responses to some of the measurements, leaving 231 people who gave consent.

The average age of the participating patients was 42 years (SD 10, range 19–66 years), and 53% were female. The subjects underwent a clinical examination and included patients with painful conditions with different ICD 10 diagnoses in chapters M00-M99. All included patients had longstanding musculoskeletal pain (> 6 months; 90% had had pain for more than one year and 23% for more than 10 years). Low back and leg, neck-, shoulder and arm pain, and multiple pain sites comprised 47%, 31% and 22% of the population, respectively. Twenty per cent had primary school education, 40% had vocational training, 12% had high school education and 28% had college/ university education. At the time of the study, 32% were working, 33% were on sick leave, and 35% were in a rehabilitation program, disability or age pension. The consenters had a higher educational level (Primary school education in non-consenters 36%, and College/University 24% in non-consenters and included more men (47% versus 24% among non-consenters). The study was approved by the Norwegian Regional Committee for Medical Research

### Measures

The Pain Stages of Change Questionnaire (PSOCQ) is a measure of individuals’ readiness to adopt a self-management approach to chronic pain conditions [6]. PSOCQ is composed of 30 items grouped in four distinct scales that represent the four stages of change from the Transtheoretical Model of behaviour change: Precontemplation (7 items; belief that management of the pain problem is primarily the responsibility of medical professionals), Contemplation (10 items; consideration of adopting a self-management approach, but reluctant to give up a medical solution), Action (6 items; beginning attempts to improve self-management skills), and Maintenance (7 items; commitment to pain self-management). Each item is accompanied by a 5 point Likert scale with scoring alternatives ranging from “strongly disagree” [1] to “strongly agree” [5]. The validated Norwegian version of PSOCQ was used [19]. Only two subjects had missing items in PSOCQ which were handled by the Rasch analysis [20].

### Pain intensity

Pain intensity during activity was measured for the last week by numeric rating scale (NRS) from 0 (no pain) to 10 (worst pain imaginable).

### Hopkins symptoms check list – 25 (HSCL-25)

Psychological distress was assessed by the Norwegian version of Hopkins symptoms check list version 25 (HSCL-25). Validity of the instrument for assessing dimensions of psychological distress has been found in several studies [21,22]. The questionnaire contains 25 questions comprising the dimensions of depression, anxiety and somatisation. The items are scored on a 4 points Likert scale ranging from not at all [1] to very much [4]. The scores of the items are summed and then divided by 25. This gives a possible total score range for HSCL-25 from 1.0 to 4.0. The cut off score for HSCL-25 is suggested to be 1.70 [22]. A higher score indicates a higher level of emotional distress. The subjects mean score in HSCL substituted occasional missing items in individual subjects according to the recommendations [23].

### Arthritis self-efficacy scale (the self-efficacy for pain subscale)

Self-efficacy was assessed using the subscale of pain in the Arthritis Self-Efficacy Scale (ASES) [24]. The instrument has been validated for a Swedish population [25], and the Norwegian version of the ASES self-efficacy for pain subscale has been used in several studies on back pain [26,27]. The scoring options used a Likert scale ranging from “totally disagree” (0) to “totally agree” [10]. The raw scores for the 5 items are summed and then divided by 5, giving a possible range from 0–10. A higher score indicates a higher degree of self-efficacy for pain.

### Data analysis and statistics

Descriptive statistics with mean, SD and frequencies were applied to describe the patient population. The people who gave consent and those who did not were compared with independent sample t-tests and the Chi-square tests (gender and education). For the Rasch analysis, age was dichotomised according to the median age (42 years), and education was dichotomised into the groups 12 years and below and higher education (above 12 years).

The Rasch analysis, partial credit model, was applied for PSOCQ and its four subscales, as the likelihood ratio test indicated lack of fit to an interval model (p < 0.001). This model is valid without the assumption of equidistance between the scoring options for each item (I) [28]. The ordering of scores for each item was examined, and scores with overlapping thresholds collapsed. Local response dependency of the items was evaluated by correlation of the residuals of the categories, with a coefficient of 0.3 as threshold value. Negative correlations between the residuals indicate multidimensionality.

Fit of individual persons and items was reported as a mean logit with SD, a mean logit of 0 and a SD of 1 representing an optimal fit of the items. The fit of the items was statistically evaluated by standardised residuals and Chi-square statistics according to the Weighted Maximum Likelihood Method [29]. Residual values within ±2.5 and a non-significant Chi-square probability value were considered to indicate adequate fit to the Rasch Model of each item [30].

To study the overall fit of items and persons to the Rasch model of the PSOCQ and its subscales the Chi-square item trait interaction statistics was applied [30]. A non- significant probability value supports fit to the model. Verification of unidimensionality was undertaken by creating two subsets of items representing the items with the most positive and most negative residuals according to a Principal Component Analysis. Person estimates for each of the two subsets were calculated, and compared by paired sample t-tests [31]. Similar estimates indicate unidimensionality of the underlying construct. The percentage of t-tests with p values below 0.05 and the corresponding Confidence interval (CI) were reported. The recommendation for a unidimensional construct is that CI should include 0.05.

Differential Item Functioning analyses (DIF). is assessed by analysis of variance for each item, comparing scores across each level of age, gender, and education [16]. Significant main effects (uniform DIF), interaction (non-uniform DIF) and subgroups of the patients (class intervals according to the level of readiness for self-management) were evaluated. Improvement of fit by split of the items with DIF was explored before removing items. The F ratio (F) for the group difference and probability (p) were given for the DIF analysis. To provide information about how the item captures the different levels of readiness to self-management, the hierarchical distribution and location (log value) of the items and persons are reported for the subscales. A scale is perfectly targeted when the mean of the persons is similar to the mean of the items. The Person Separation Reliability Index (PSI) is reported, providing an indication of consistency of items, and thus the power to discriminate among persons with different levels of readiness to self-management. The PSI is comparable to Cronbachs alpha but with a linear Rasch based transformation of scores [32]. A value above 0.8 was deemed to differentiate across at least 3 patient groups [30]. Subtest analysis was conducted with each subscale representing one item (superitem) in the Rasch analysis. The sumscores of the Precontemplation, Contemplation, Action and Maintenance subscales were calculated. The Rasch calibrated person estimates based on the items fitting the Rasch model in each subscales were also calculated.

The Rasch analysis was performed in RUMM 2030 (RUMM laboratory, Perth, Australia). Other analyses were performed by SPSS for Windows version 18.0. A significance level of 0.05 was adopted. This significance level was Bonferroni corrected, according to the number of items and groups tested [33].

## Results

The 30 items in the PSOCQ did not fit the Rasch model as indicated by the Chi-square item trait statistics (X^2^ = 198, p < 0.0001). The fit of the residuals for the items was 0.44, SD 0.73 and the fit of the persons -0.40, SD 2.01, which indicate misfit of the persons to the model. The threshold pattern for the scores of the items was disordered for twelve of the items in PSOCQ. The scores had to be collapsed in order to obtain distinguishable thresholds along the trait. However, correlation analysis of residuals revealed extensive local response dependency (correlation coefficient >0.3) and also substantial negative correlation coefficient indicating multidimensionality. Rescoring of the thresholds for the twelve items, removal of items with correlation coefficients >0.3 and sequential removal of the items with the highest negative correlation coefficients of residuals did not obtain a solution with fit to the Rasch model. The Principal Component Analysis revealed four factors with Eighen value above 1.5. Hence, the items were split into the four subscales and reanalysed.

### Precontemplation

Two of the seven items displayed disordered thresholds and categories had to be collapsed (Table 1). There was no local response dependency. The item *I12 My pain is a medical problem and I should…, I22 I still think despite what doctor tell me..*and *I24 The best thing I can do is to find a doctor..* did not fit the model and were removed (fit residual >2.5). A slight DIF by age was found for the item *I25Why can’t someone just do something…,* with subjects above 42 years reporting higher levels than expected by their overall level of Precontemplation. However, as this DIF did not cause significant misfit of the item, and split of the item did not improve fit, it was kept without split in the subscale. The overall

**Figure 1:**
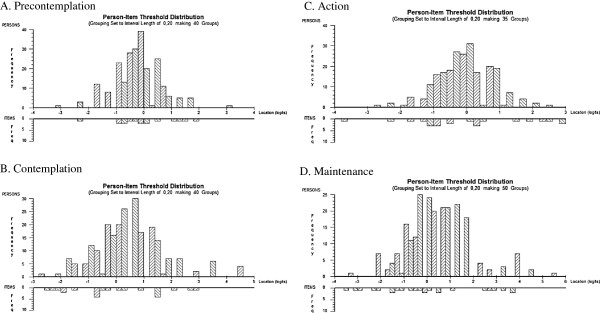
**The distribution of the items in precontemplation (A), contemplation (B), action (C) and maintenance (D) subscales and the patients (N = 231) along the Rasch calibrated metric scale.** The upper panel shows the location of the subjects and the lower panel the threshold of the items.

Chi-square statistics was 16.48, df =12, p = 0.17 indicating fit to the Rasch model. Person estimates were calculated for the most positively loaded and the most negatively loaded items. PCA of the residuals of these two estimates did not differ significantly as evaluated by the *t*-test (1.20%, CI 0.50% to 4.63%). PSI was 0.50, which indicates rather low internal consistency, and there were negative residual correlations below -0.3 between three of the items indicating some remaining multidimensionality. The mean person location was -0.20 (SD 0.78) indicating adequate targeting as well as distribution of the threshold for the scores of the items patients (Figure 1A).

### Contemplation

Four items had disordered thresholds and were rescored (Table 1). No DIF by age, gender or education was found. Item *I8 Even though my pain is not going away…* and *I9 I have realised now that it is time for me to come up..* showed local response dependency with a residual correlation of 0.36 and the latter item also misfitted the model and was removed. . Item *I15 I have recently figured out that it is up to me..,*, *I21 I am starting to wonder whether it is up to me..* and *I23 I have been thinking that doctors can only help..* misfitted the model (fit residual outside ±2.5). No DIF could explain this misfit and guide split of the items, hence these items were removed. The remaining seven items fitted the model (Table 2), and the item trait interaction statistics (X^2^ = 12.15, df = 18, p = 0.84) was not significant. PSI was 0.77. The *t*-test supported unidimensionality (5.63%, CI 2.66% to 8.60%). The mean person location was 0.49 (SD 1.24) indicating slightly higher contemplation ability among subjects compared to the items, however with a well distribution of the threshold for the scores (Figure 1B).

### Action

One item was rescored due to disordered threshold (Table 1). No local response dependency or DIF was found for the items in this scale which all fitted the Rasch model (Table 2) and revealed a non-significant item trait statistics (X^2^ = 19.88, df = 18, p = 0.34). However, PSI was 0.59 and rather low. The *t*-test supported unidimensionality (5.26% CI 2.36% to 8.16%).

Mean location of persons was -0.07 (SD 0.80) indicating rather good targeting of the trait and distribution of thresholds for the items (Figure 1C).

### Maintenance

One item was rescored (Table 1). No local response dependency or DIF was found. *I13 I am beginning to wonder if I need some help*…, misfitted the model and was removed.

The remaining six items fitted the model (Table 2), and also the overall fit statistics was non-significant (*X*^2^ = 10.88, df = 18, p = 0.92. PSI was 0.80, and the *t*-test supported unidimensionality (6.49% CI 3.03% to 9.50%). Mean location of persons was 0.41 (SD 1.35) indicating a slight offset with the subjects capturing slightly higher level of maintenance than the items. There was also a gap in the distribution of thresholds for the items (Figure 1D).

**Table 1:** Items (I) with disordered thresholds in the four subscales and the subsequent collapsed score options

**PSOQ items**	**Revised scores**
*Precontemplation*	
I12. My pain is a medical problem and I should..	1,1,2,3,4
I22. I still think despite what doctor tell me..	1,1,2,3,3
*Contemplation*	
I7. I have recently realised that there is no medical cure…	1,1,2,3,4
I14. I am beginning to wonder if I need some help..	1,1,2,3,4
I21. I am starting to wonder whether it is up to me…	1,1,2,3,4
I23. I have been thinking that doctors can only help…	1,1,2,3,4
*Action*	
I20. I am getting help learning some strategies for..	1,2,3,3,4
*Maintenance*	
I13. I am currently using some suggestions people …	1,2,3,3,4

**Table 2:** Fit of the items in the subscales of the precontemplation (A), contemplation (B), action (C) and maintenance (D) subscale according to the Rasch analysis (n = 231)

**Item**	**Content**	**Location**	**SE**	**Residual**	**χ2**	**Prob,.**
A. Precontemplation						
I 11	I have tried everything that people recommended…	-0.39	0.07	1.47	2.54	0.47
I 16	Everyone I speak with tells me I have to learn..	0.06	0.07	0.35	3.93	0.27
I 25	Why can’t someone just do something..	-0.35	0.06	-0.66	6.60	0.09
I 29	I have been wondering if there is something I..	0.68	0.08	1.44	3.41	0.33
Bonferroni corrected significance level 0.01						
B. Contemplation						
I 1	I have been thinking that the way I cope with my pain..	0.26	0.09	0.99	2.61	0.46
I 7	I have recently realised that there is no medical cure…	0.29	0.09	1.06	0.38	0.94
I 8	Even though my pain is not going away…,	-0.80	0.09	-1.47	7.06	0.07
I 14	I am beginning to wonder if I need some help..	0.38	0.09	1.12	0.73	0.87
I 19	All this talk about how to cope better..	0.51	0.09	0.54	0.09	0.99
I 28	I have been wondering if there is something I..	-0.65	0.10	0.06	1.28	0.73
Bonferroni corrected significance level 0.008						
C. Action						
I 2	I am developing new ways to cope with my pain	-0.52	0.09	-1.11	4.74	0.19
I 6	I have started to come up with some strategies..	-0.40	0.09	-0.10	3.54	0.32
I 20	I am getting help learning some strategies for..	0.82	0.09	2.19	2.41	0.49
I 26	I am learning to help myself controlling my pain without..	0.16	0.08	1.32	1.43	0.70
I 27	I am testing out some coping skills to manage..	0.05	0.08	-0.40	6.06	0.11
I 30	I am learning ways to control my pain without..,	-0.03	0.07	0.55	1.71	0.63
Bonferroni corrected significance level 0.008						
D. Maintenance						
I 3	I have learned some good ways to keep my pain	0.30	0.09	1.27	0.24	0.97
I 4	When my pain flares up I find myself automatically..	0.16	0.08	0.73	1.09	0.78
I 5	I use some strategies that help me better deal with..	-0.36	0.10	-1.13	6.20	0.10
I 10	I use what I have learned to keep my pain..	-0.56	0.10	0.64	0.43	0.93
I 17	I have incorporated techniques..	-0.28	0.10	-1.87	1.35	0.72
I 18	I have made a lot of progress in coping with my pain	0.74	0.09	1.05	1.09	0.78
Bonferroni corrected significance level 0.008						

### Subtest analysis

Subsequently a subtest analysis of the whole PSOCQ scale was performed by creating subtest of the fitting items in each subscale. Overall fit to the Rasch model was not found (*X*^2^ = 47.71, p < 0.001), with local response dependency of 0.40 between the Action and Maintenance scale and misfit of the Precontemplation subscale.

### Relationship between subscales and pain, emotional distress and self-efficacy

The Rasch calibrated person estimates for the Precontemplation subscale kept its external validity indicating that high level of the Precontemplation trait was associated with higher pain during activity, higher emotional distress and lower self-efficacy. Less consistent associations were found for the other subscales. The positive association between the self-efficacy and the Maintenance sucscale, while the association to the Action subscale is worth noting. Furthermore, the reduced number of items in the Rasch adapted subscales did not alter the associations (Table 3).

**Table 3:** Pearson correlation coefficient and p-value for the association between the PSOCQ subscales and pain during activity, emotional distress and self-efficacy

**Subscales**	**Pain during activity**	**Emotional distress**	**Self-efficacy**
Precontemplation	r = 0.29,p < 0.001*	r = 0.34, p < 0.001*	r = -0.25, p < 0.001*
	(r = 0.32,p < 0.001)	(r = 0.32, p < 0.001)	(r = -0.27, p < 0.001)
Contemplation	r = 0.04,p = 0.59	r = 0.18, p = 0.08	r = -0.05, p = 0.46
	(r = 0.06,p = 0.36)	(r = 0.14, p = 0.04)	(r = -0.12, p = 0.09)
Action	r = 0.08,p = 0.90	r = -0.15, p = 0.02	r = -0.28, p < 0.001*
	(r = -0.09,p = 0.90)	(r = -0.17, p = 0.01)	(r = -0.27, p < 0.001)
Maintenance	r = -0.04,p = 0.57	r = -0.19, p = 0.04	r = 0.22, p = 0.001*
	(r = -0.03,p = 0.71)	(r = -0.17, p = 0.01)	(r = 0.19, p = 0.005)

The results based on the Rasch calibrated person estimates for the revised subscales are reported with the results for the original uncalibrated sumscores in parenthesis.

*Bonferroni corrected significance level 0.02.

## Discussion

The present Rasch analysis did not support our explorative hypothesis of one unidimensional construct of readiness to self-management of pain. Consistent with results obtained by factor analysis in previous studies, the PSOCQ had to be revised and divided in subscales. Also in accordance with previous factor analysis showing close relationship between the Action and Maintenance subscales [14], the present results indicated the possibility of combining these subscales.

Identifying readiness to self-management and adhering to rehabilitation is of importance in pain management [34]. Within this framework, the PSOCQ, with its four subscales, has been developed and applied [6]. The number and content of subscales have been subject to discussion [9,12,14,19]. The inconsistent results may partly be flawed by applying statistics assuming scaling properties on the ordinal measurement PSOCQ [35]. Furthermore, based on the poor predictive value of the subscales in PSOCQ regarding pain recovery, alternative strategies to single out predictive factors from PSOCQ including creating cluster profiles, have evolved [13]. Hence, applying modern measurement theory i.e. Rasch analysis not assuming interval scaling of the responses may provide new insight into the dimensionality of PSOCQ.

An important requirement is that the measurement should represent a single underlying construct [30,36]. Further, if reflecting several constructs, each construct should be summed up in a separate subscale. The PSOCQ is based on the TTM for behavioural change, and theoretically reflects four stages of change (the four subscales). Hence, as expected, all 30 items in PSOCQ could not be fitted in a single construct in the present analysis. The Principal Component Analysis which is a factor analysis but based on Rasch corrected instead of raw scores as recommended for ordinal measurements [35]. Furthermore, correlations of the residuals from the Principal Component analysis were evaluated. The positive correlations among the residuals of the items in PSOCQ indicate that the responses to the items are not independent of each other [37]. Independent responses to each item, is a necessary requirement for a measurement, and lack of independency may inflate the judged reliability of the scale.

The positive residual correlation between the Action and Maintenance subscales revealed in the subtest analysis indicate local dependency between these subscales and may favour a collapse of these subscales. This is in agreement with previous studies identifying three factors in PSOCQ [9]. The lack of fit of the four subscales to a common construct in the subtest analysis support the notion of separate phenomenon reflected by the subscales of PSOCQ.

The Precontemplation and Contemplation items fitted the Chi-square item trait statistics after the removal of three and four items, respectively. The items contained in the Precontemplation subscales reflect that the person still seeks medical or external solutions to the pain. However, even after rescoring and removing misfit items several negatively residual correlations between the items were found in the Precontemplation subscale. Negative correlation coefficients indicate multidimensionality [31]. Furthermore, internal consistency of the items as evaluated by the PSI was far from optimal. PSI is equivalent to Cronbachs alpha but can be calculated with missing values. The present low value implies that the scale cannot differentiate between subjects with different levels of the Precontemplation trait [30], which is important from a clinical perspective. This unsatisfactory internal consistency may be caused by slight differences in underlying constructs of the items supported by the negative residual correlations of these items. The finding of multidimensionality in the Precontemplation subscale is not surprising, as the individual items reflect different characteristics like believe in medical solution, lack of pain control, and fear of movement. Possibly, rephrasing of some of the items may be needed in order to reduce misfit, but was beyond the scope of this study. On the other hand, the four items covered the distribution of the Precontemplation trait in the persons and the subscale was well targeted. The Precontemplation has been considered to have the best predictive value for poor treatment outcome among the subscales [38]. Even with only four retained items, the association to pain, emotional distress and self-efficacy was kept by the revised Precontemplation subscale.

The items in the Contemplation subscale indicate considerations of starting to use pain self- management techniques. The seven items fitting the Rasch model in this subscale revealed far better intrinsic measurement properties than the Precontemplation items, with the ability to differentiate between two levels of the contemplation trait as evaluated by the PSI trait [30]. The Contemplation subscale had weaker relationship to pain, emotional distress and self-efficacy than the Precontemplation subscale in accordance with several previous studies [9,12,19]. This weaker relationship along with the lack of fit in the subtest may be a reason to reconsider the necessity of keeping the Contemplation subscale in the PSOCQ.

The measurement properties of the Action and Maintenance subscales were rather good after slight modifications of included items and scores. The close association between these subscales may represent a possibility for collapse and reduction in the number of items. The negative correlation between Action and self-efficacy, while the Maintenance trait was positively associated with self-efficacy could contradict this conclusion. Further studies investigating the possibility of characterising subjects according to the Precontemplation and Maintenance subscale is advocated.

At last, the items in the subscales of PSOCQ were robust to variance according to important factors like age, gender and education, which is important to be able to sum the scores in persons with different age, education and gender. Of course, this is also a prerequisite to the validity of the scale.

A body of literature indicates that many commonly used ordinal scales may not meet the requirement of interval scaling necessary to calculate change scores of a measurement [39,40]. The scaling properties can be evaluated by Rasch analysis [36]. Regarding PSOCQ, more that 30% of the items had to be rescored in order to provide valid and distinct score options, and several items had to be excluded due to misfit to the subscale construct.

Improving outcome measurements suited for rehabilitation in patients with chronic pain is important. Rasch analysis provides the possibility to assess the unidimensionality of the underlying construct, and also provide Rasch calibrated scores that can be used to calculate change scores and effect sizes [37]. However, applying Rasch analysis to an existing measurement does not raise the possibility of including new items or rephrase them. In addition, the present population was Norwegian and characterised by chronic musculoskeletal pain. Hence, differences across pain conditions and cultures will need to be assessed.

## Conclusion

The present analysis revealed that all four subscales in PSOCQ fitted the Rasch model when misfit items were removed. No common construct for all subscales were identified, but the Action and Maintenance subscale were closely related. Invariance indicates applicability across subgroups of patients, but internal consistency was low for the Precontemplation and Action subscales. The Precontemplation subscale showed the strongest association to pain, emotional distress and self-efficacy.

## Competing interests

The authors declare that they have no competing interests.

## Authors’ contributions

All four authors have contributed to the design, preparation of manuscript and have approved the final version. ED and TF have provided main contribution to data collection, CR, ED and AA have been the main contributors in the data analysis.

## Pre-publication history

The pre-publication history for this paper can be accessed here:

http://www.biomedcentral.com/1471-2474/15/95/prepub
